# Supplementary material for: Advanced parametrization for the production of high-energy solid-state lithium pouch cells containing polymer electrolytes
Source: Nat Commun. 2024 Jul 12;15:5860. doi: 10.1038/s41467-024-50075-9 (PMC11245499; doi:10.1038/s41467-024-50075-9)
Supplement: Supplementary file 1 — Supplementary Information [file 41467_2024_50075_MOESM1_ESM.pdf]

Supplementary Information

Advanced parametrization for the production of high-energy solid-state lithium pouch cells containing polymer electrolytes

Wonmi Lee[a,†], Juho Lee[a,b,†], Taegyun Yu[a,†], Hyeong-Jong Kim[b], Min Kyung Kim[a,c], Sungbin Jang[a], Juhee Kim[a], Yu-Jin Han[a], Sunghun Choi[d,e], Sinho Choi[a], Tae-Hee Kim[a], Sang-Hoon Park[a], Wooyoung Jin[a], Gyujin Song[a], Dong-Hwa Seo[f], Sung-Kyun Jung[*,b], Jinsoo Kim[*,a]

[a] Ulsan Advanced Energy Technology R&D Center, Korea Institute of Energy Research, Ulsan, Republic of Korea

[b] Department of Energy Engineering, School of Energy and Chemical Engineering, Ulsan National Institute of Science and Technology (UNIST), Ulsan, Republic of Korea

[c] Department of Nano Fusion Technology, Pusan National University, Busan, Republic of Korea

[d] Gwangju Clean Energy Research Center, Korea Institute of Energy Research, Gwangju, Republic of Korea

[e] Department of Battery Convergence Engineering, Kangwon National University, Chuncheon, Republic of Korea

[f] Department of Materials Science and Engineering, Korea Advanced Institute of Science and Technology (KAIST), Daejeon, Republic of Korea

* Email addresses of corresponding authors.

Tel: +82-52-702-2534, jkim@kier.re.kr (J. Kim)

Tel: +82-52-217-3030, skjung@unist.ac.kr (S.-K. Jung)

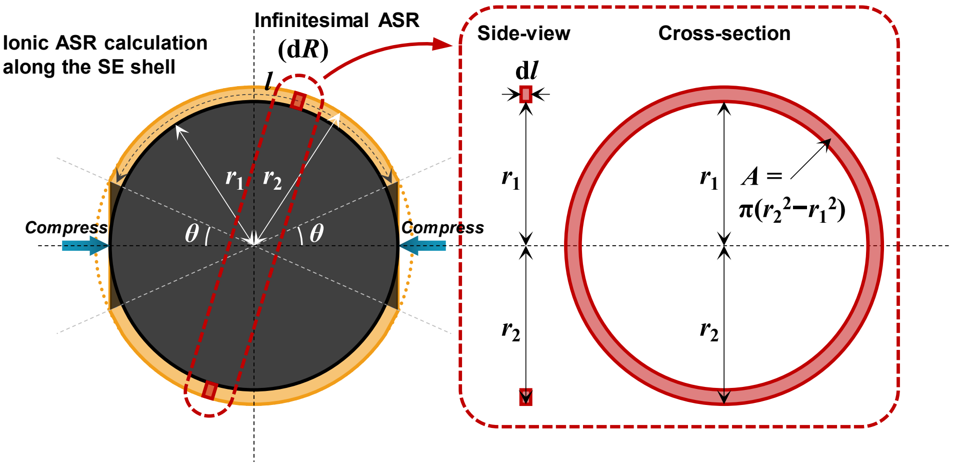

Supplementary Fig. 1: Schematic of ASR representation in SE shell with infinitesimal component.

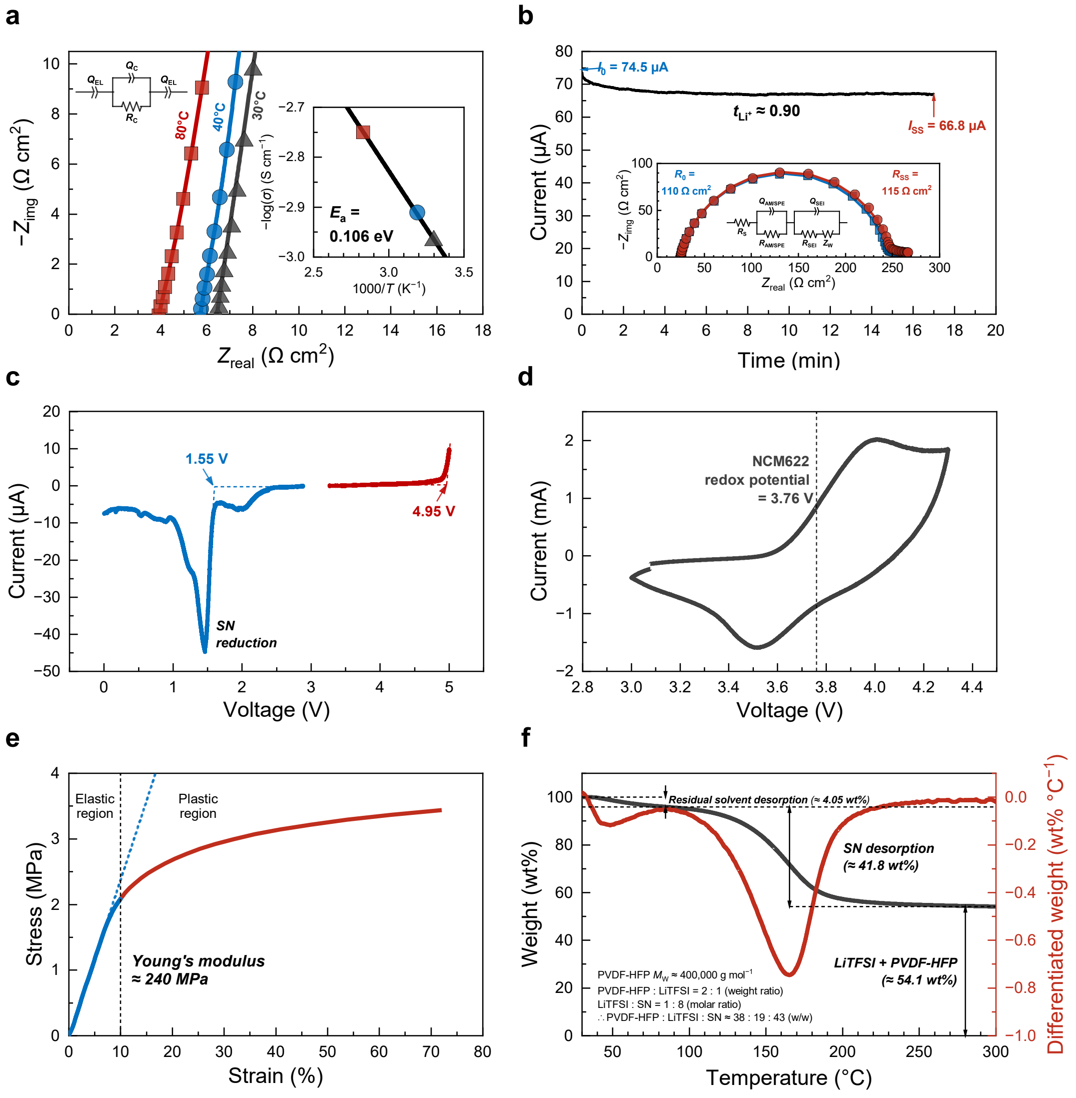

Supplementary Fig. 2: Properties and electrochemical performance of freestanding solid polymer electrolyte. a EIS data of SS|SPE|SS coin cell and Arrhenius plot for calculation of the activation energy of the SPE at 30°C, 40°C and 80°C. **b** EIS and CA results of Li|SPE|Li coin cell for calculation of the lithium-ion transference number at 25°C. **c** LSV curve of Li|SPE|SS coin cell to show electrochemical stable window at 25°C. **d** CV curve of Li|SPE|NCM622 coin cell to show the redox potential of NCM622 as an AM in the positive electrode at 25°C. **e** DMA result showing the mechanical strength of the SPE. **f** TGA curve showing the thermal stability of the SPE.

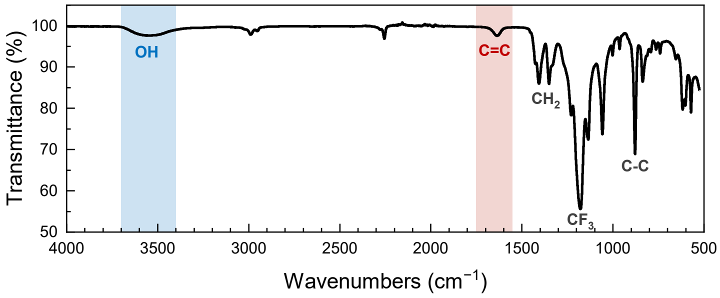

Supplementary Fig. 3: FTIR data of SPE (consisting of PVDF-HFP as the polymer matrix, LiTFSI as the Li salt, and SN as a plasticizer).

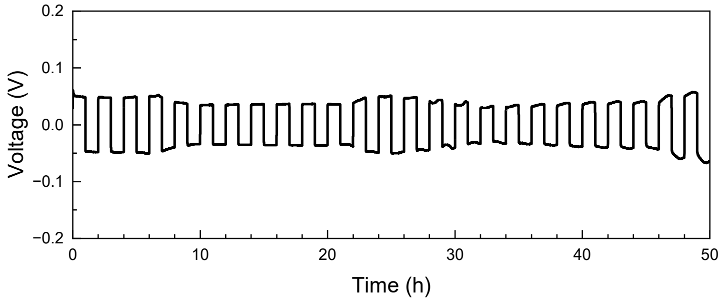

Supplementary Fig. 4: Li symmetric cycling result. Li|SPE|Li coin cell with current density of ±0.15 mA cm^{-2} for 1 h as each cycle at 25°C.

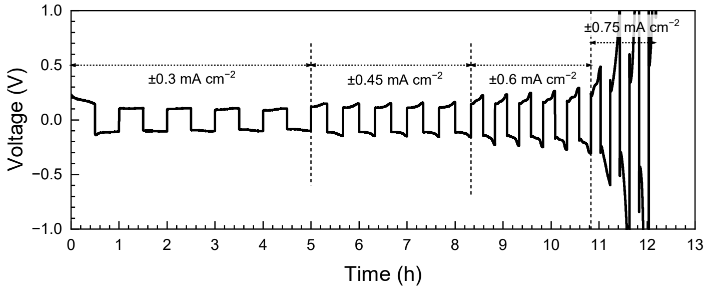

Supplementary Fig. 5: Li symmetric critical current density test. Li|SPE|Li coin cell with constant areal capacity (0.15 mAh cm^{-2}) and different current densities (±0.3 mA cm^{-2} for 30 mins (for 1-5 cycles), ±0.45 mA cm^{-2} for 20 mins (for 6-10 cycles), ±0.6 mA cm^{-2} for 15 mins (for 11-15 cycles), and 0.75 mA cm^{-2} for 12 mins (for 16-19 cycles) at 25°C.

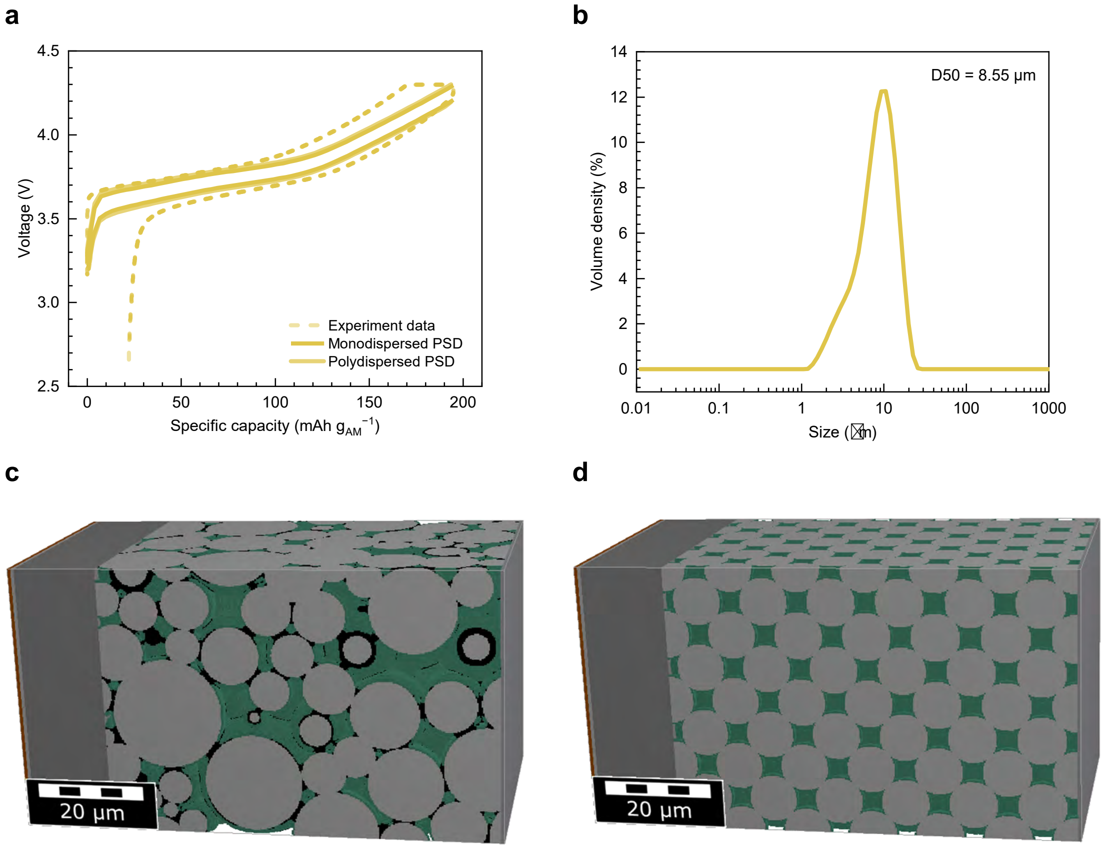

Supplementary Fig. 6: Comparison of the electrochemical characteristics of SSB pouch cell and the corresponding simulated monodispersed and polydispersed PSD models. a the electrochemical profile in **Fig. 6a** and the simulated profile from corresponding PSD models, **b** the particle size distribution of NCM622 used in the modeling, and the visualized electrode structure for **c** polydispersed and **d** monodispersed PSD models.

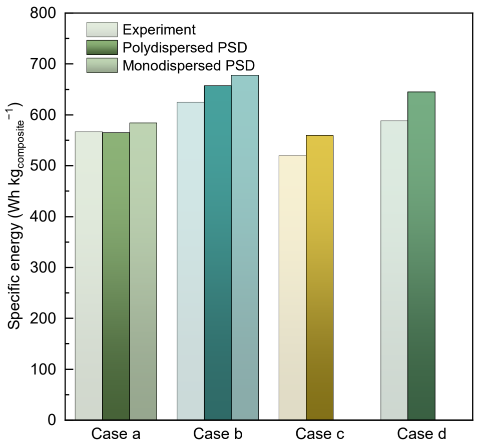

Supplementary Fig. 7: Specific energy comparison divided by composite for each case. Case a-b are well-percolated cases and Case c-d are poor-percolated cases in each AM-poor and AM-rich conditions, respectively.

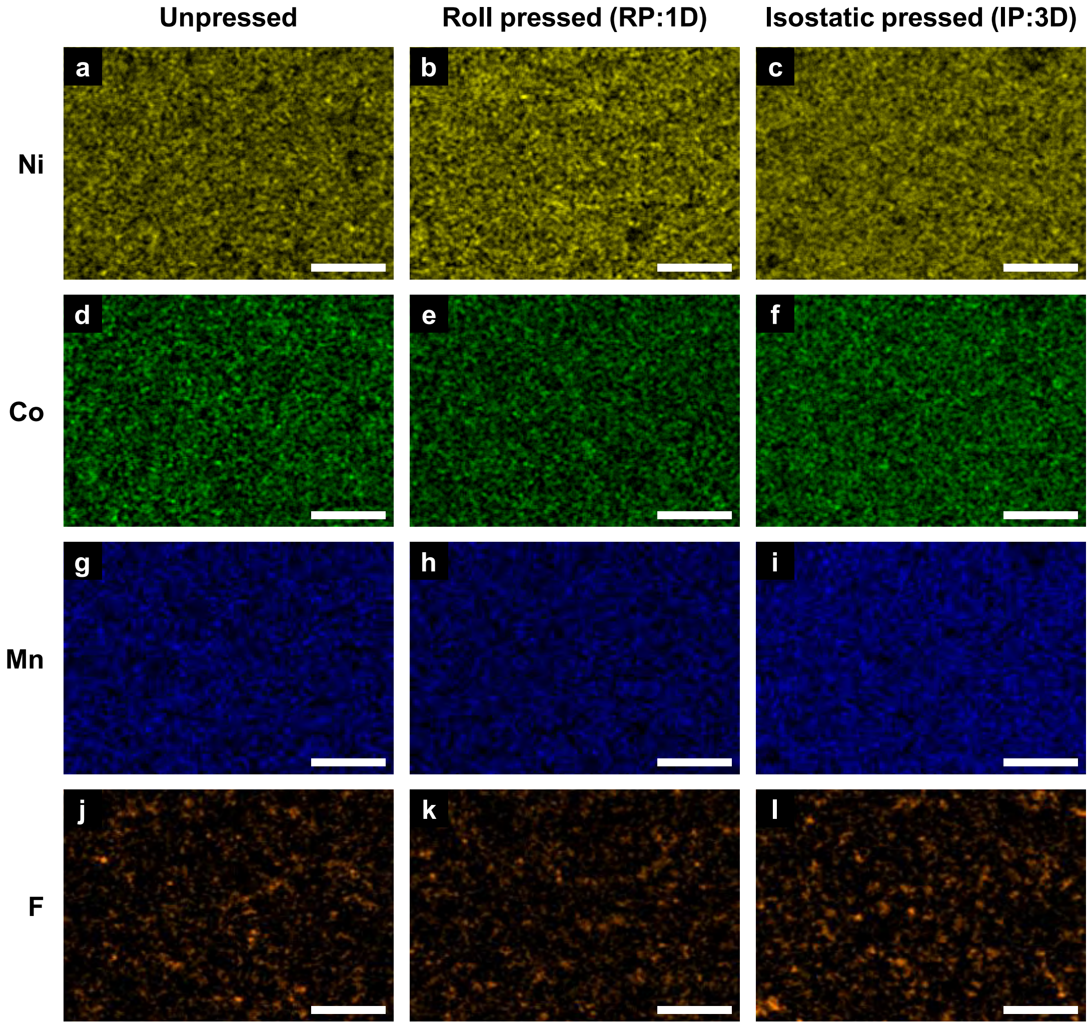

Supplementary Fig. 8: EDS data of positive electrodes prepared using different pressing methods. **a, d, g, j** unpressed, **b, e, h, k** roll-pressed, and **c, f, i, l** isostatic-pressed methods while represent **a-c** Ni, **d-f** Co, **g-i** Mn, **j-l** F. The scale bars are 100 μm.

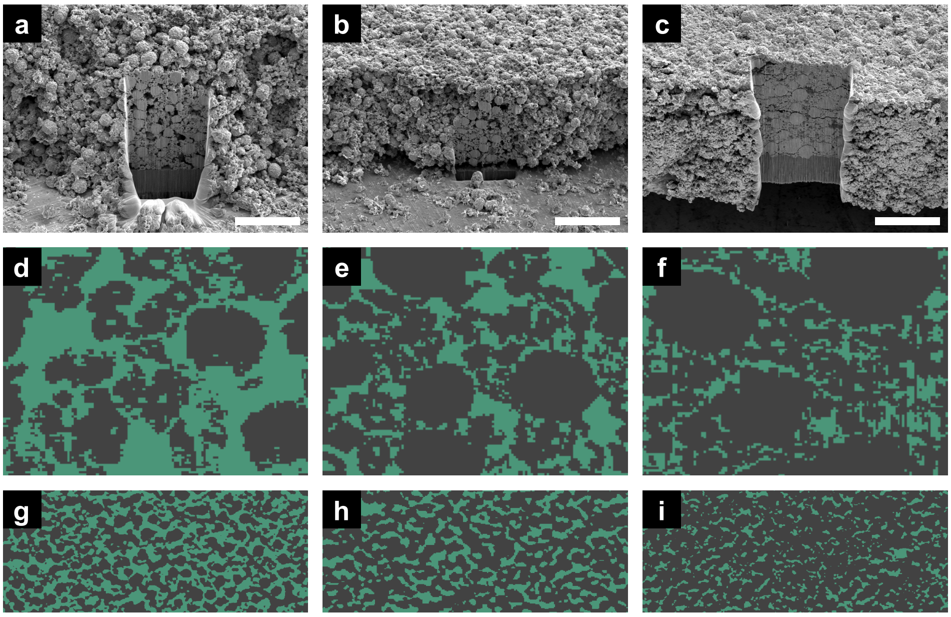

Supplementary Fig. 9: 3D and 2D FIB and X-ray CT images of the positive electrodes prepared using different pressing methods. 3D view of FIB image of positive electrodes prepared using **a** unpressed, **b** roll-pressed, and **c** isostatic-pressed methods. 2D FIB images of positive electrodes prepared using **d** unpressed, **e** roll-pressed, and **f** isostatic-pressed methods (the green and black colors indicate voids and AM, respectively.). 2D X-ray CT image of positive electrodes prepared using **g** unpressed, **h** roll-pressed, and **i** isostatic-pressed methods (the green and black colors indicate voids and AM, respectively. The scale bars are 50 μm.).

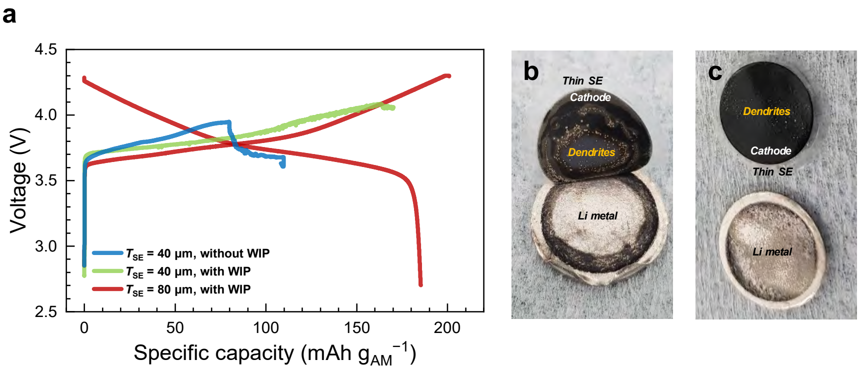

Supplementary Fig. 10: Coin-cell performance of SSBs using different thicknesses of SE membranes. a Charge–discharge curves of Li|SPE|NCM622 coin cell with thin SE membrane (40 μm) without WIP, thin (40 μm) and thick (80 μm) SE membranes compressed with WIP at 25°C. b, Photo of disassembled coin cells with 40 μm thick SE membrane without **b** and with **c** WIP.

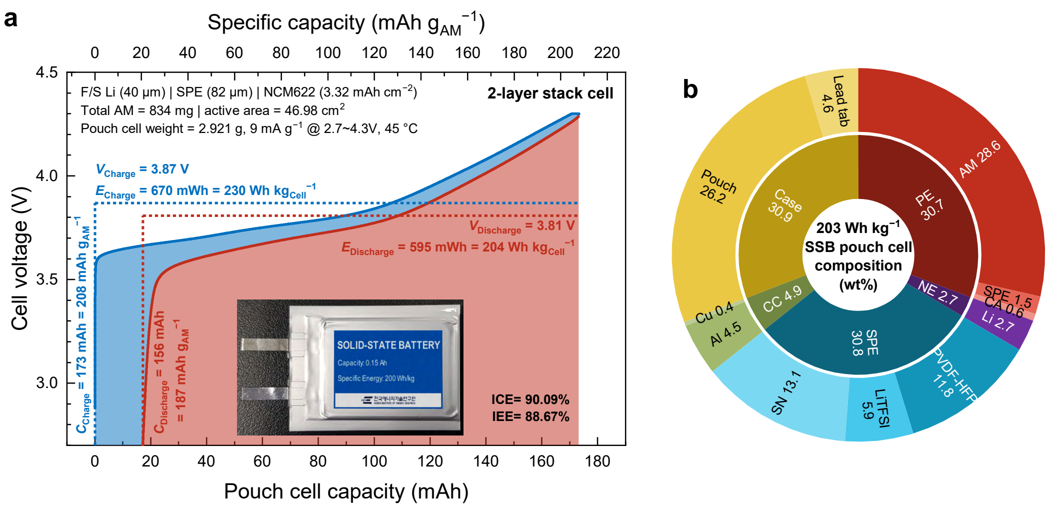

Supplementary Fig. 11: Electrochemical characteristics and compositions of practical SSB pouch cells. First charge–discharge electrochemical profiles at 25°C and weight compositions of all the configuring materials of SSB pouch cells with **a–b** 156 mAh scale, 200 Wh kg^{-1}

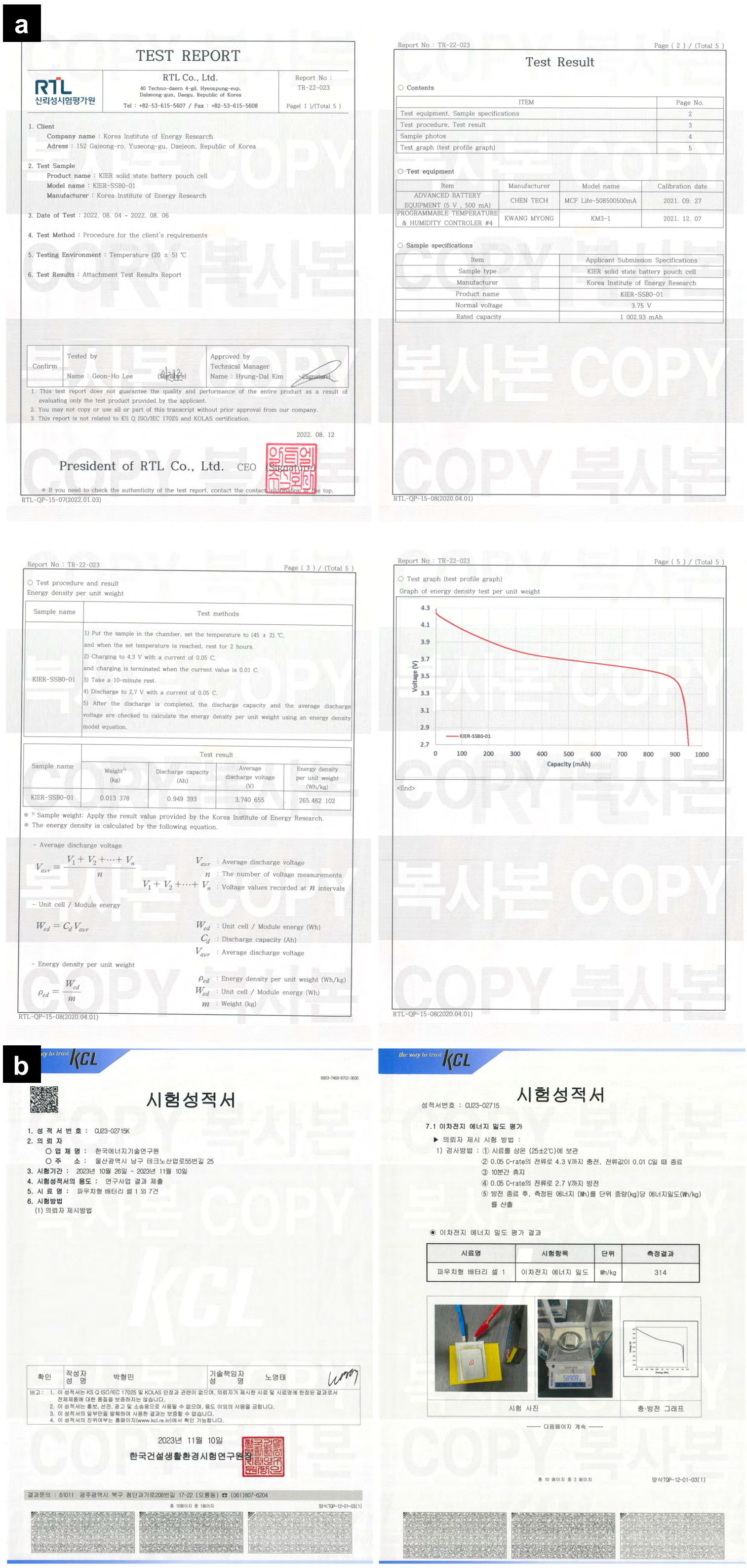
a

TEST REPORT

RTL Co., Ltd.
40 Techno-daero 4-gil, Hyeonpung-eup, Dalseong-gun, Daegu, Republic of Korea
Tel : +82-53-615-5607 / Fax : +82-53-615-5608

Report No : TR-22-023
Page(1)/(Total 5)

1. Client
Company name : Korea Institute of Energy Research
Adress : 152 Gajeong-ro, Yuseong-gu, Daejeon, Republic of Korea

2. Test Sample
Product name : KIER solid state battery pouch cell
Model name : KIER-SSB0-01
Manufacturer : Korea Institute of Energy Research

3. Date of Test : 2022. 08. 04 ~ 2022. 08. 06

4. Test Method : Procedure for the client's requirements

5. Testing Environment : Temperature (20 ± 5) ℃

6. Test Results : Attachment Test Results Report

Confirm — Tested by: Name : Geon-Ho Lee (Signature); Approved by: Technical Manager, Name : Hyung-Dal Kim (Signature)

1. This test report does not guarantee the quality and performance of the entire product as a result of evaluating only the test product provided by the applicant.
2. You may not copy or use all or part of this transcript without prior approval from our company.
3. This report is not related to KS Q ISO/IEC 17025 and KOLAS certification.

2022. 08. 12

President of RTL Co., Ltd. CEO

RTL-QP-15-07(2022.01.03)

Report No : TR-22-023 — Page (2) / (Total 5)

Test Result

○ Contents

ITEM	Page No.
Test equipment, Sample specifications	2
Test procedure, Test result	3
Sample photos	4
Test graph (test profile graph)	5

○ Test equipment

Item	Manufacturer	Model name	Calibration date
ADVANCED BATTERY EQUIPMENT (5 V , 500 mA)	CHEN TECH	MCF Life-508500500mA	2021. 09. 27
PROGRAMMABLE TEMPERATURE & HUMIDITY CONTROLER #4	KWANG MYONG	KM3-1	2021. 12. 07

○ Sample specifications

Item	Applicant Submission Specifications
Sample type	KIER solid state battery pouch cell
Manufacturer	Korea Institute of Energy Research
Product name	KIER-SSB0-01
Normal voltage	3.75 V
Rated capacity	1 002.93 mAh

RTL-QP-15-08(2020.04.01)

Report No : TR-22-023 — Page (3) / (Total 5)

○ Test procedure and result
Energy density per unit weight

Sample name	Test methods
KIER-SSB0-01	1) Put the sample in the chamber, set the temperature to (45 ± 2) ℃, and when the set temperature is reached, rest for 2 hours. 2) Charging to 4.3 V with a current of 0.05 C, and charging is terminated when the current value is 0.01 C. 3) Take a 10-minute rest. 4) Discharge to 2.7 V with a current of 0.05 C. 5) After the discharge is completed, the discharge capacity and the average discharge voltage are checked to calculate the energy density per unit weight using an energy density model equation.

Sample name	Weight[1) (kg)	Discharge capacity (Ah)	Average discharge voltage (V)	Energy density per unit weight (Wh/kg)
KIER-SSB0-01	0.013 378	0.949 393	3.740 655	265.462 102

※ 1) Sample weight: Apply the result value provided by the Korea Institute of Energy Research.
※ The energy density is calculated by the following equation.

- Average discharge voltage

$$V_{avr} = \frac{V_1 + V_2 + \cdots + V_n}{n}$$

V_{avr} : Average discharge voltage; n : The number of voltage measurements; $V_1 + V_2 + \cdots + V_n$: Voltage values recorded at n intervals

- Unit cell / Module energy

$$W_{ed} = C_d V_{avr}$$

W_{ed} : Unit cell / Module energy (Wh); C_d : Discharge capacity (Ah); V_{avr} : Average discharge voltage

- Energy density per unit weight

$$\rho_{ed} = \frac{W_{ed}}{m}$$

ρ_{ed} : Energy density per unit weight (Wh/kg); W_{ed} : Unit cell / Module energy (Wh); m : Weight (kg)

RTL-QP-15-08(2020.04.01)

Report No : TR-22-023 — Page (5) / (Total 5)

○ Test graph (test profile graph)
Graph of energy density test per unit weight

<End>

RTL-QP-15-08(2020.04.01)

b

시험성적서

1. 성 적 서 번 호 : CU23-02715K
2. 의 뢰 자
○ 업 체 명 : 한국에너지기술연구원
○ 주 소 : 울산광역시 남구 테크노산업로55번길 25
3. 시험기간 : 2023년 10월 26일 ~ 2023년 11월 10일
4. 시험성적서의 용도 : 연구사업 결과 제출
5. 시 료 명 : 파우치형 배터리 셀 1 외 7건
6. 시험방법
(1) 의뢰자 제시방법

확인 — 작성자 성명 박형민 — 기술책임자 성명 노영태

2023년 11월 10일
한국건설생활환경시험연구원장

시험성적서

성적서번호 : CU23-02715

7.1 이차전지 에너지 밀도 평가
▶ 의뢰자 제시 시험 방법 :
1) 검사방법 : ① 시료를 상온 (25±2℃)에 보관
② 0.05 C-rate의 전류로 4.3 V까지 충전, 전류값이 0.01 C일 때 종료
③ 10분간 휴지
④ 0.05 C-rate의 전류로 2.7 V까지 방전
⑤ 방전 종료 후, 측정된 에너지 (Wh)를 단위 중량(kg)당 에너지밀도(Wh/kg)를 산출

◉ 이차전지 에너지 밀도 평가 결과

시료명	시험항목	단위	측정결과
파우치형 배터리 셀 1	이차전지 에너지 밀도	Wh/kg	314

시험 사진 — 충·방전 그래프

—— 다음페이지 계속 ——

Supplementary Fig. 12: Certification of specific energy of a ~280 Wh kg^{-1} and b ~310 Wh kg^{-1} by external third-party organization.

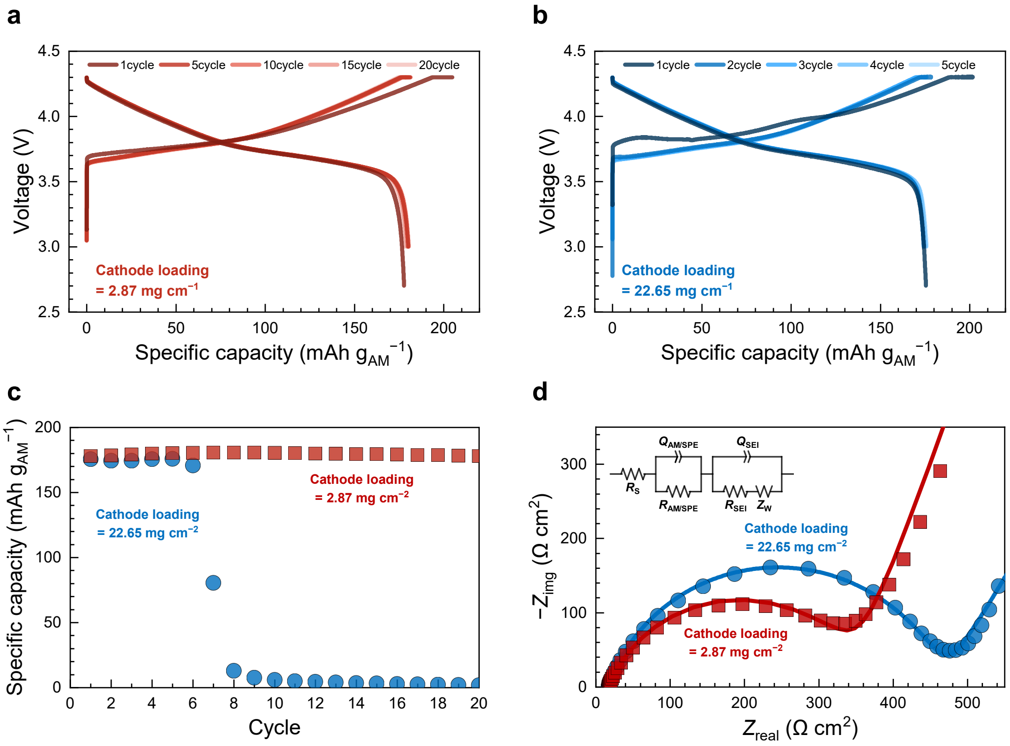

Supplementary Fig. 13: Coin-cell performance of SSBs based on SPE. a Charge–discharge curves of Li|SPE|NCM622 coin cell with low positive electrode loading (3.50 mg cm^{-2}). **b** Charge–discharge curves of Li|SPE|NCM622 coin cell with high positive electrode loading (22.67 mg cm^{-2}). **c** Discharging capacity during cycling of Li|SPE|NCM622 coin cells with different positive electrode loading levels. **d** EIS results of Li|SPE|NCM622 coin cells with different positive electrode loading levels. All tests were performed at 25°C.

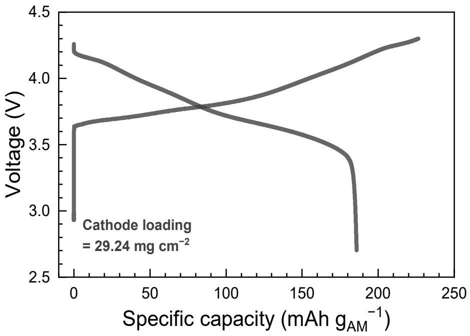

Supplementary Fig. 14: Charge–discharge curves of Li|SPE|NCM811 coin cell performance at 25°C.

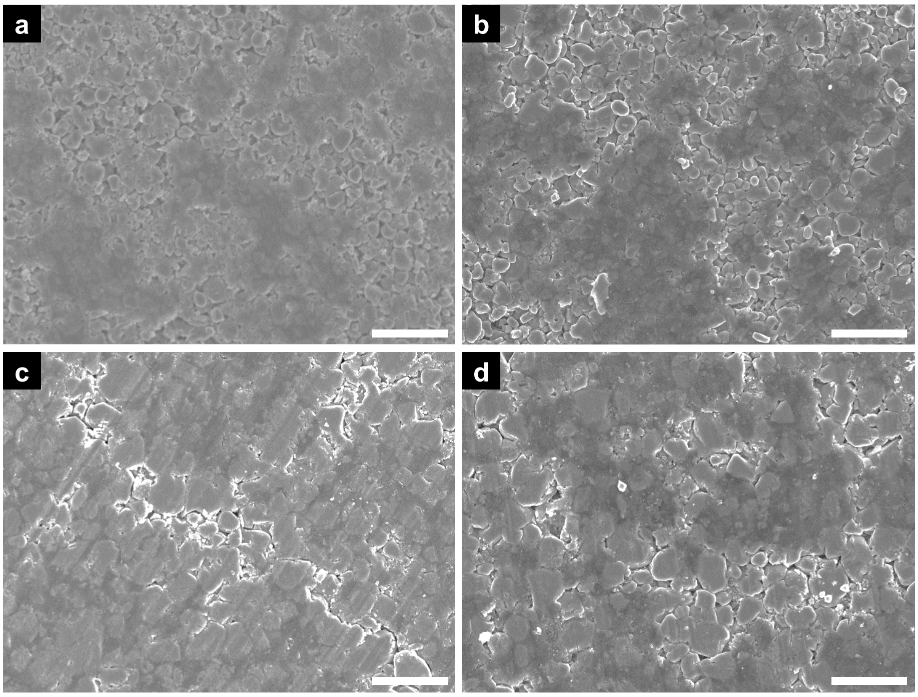

Supplementary Fig. 15: SEM images of the positive electrodes (NCM622 or NCM811) before and after Li|SPE|NCM622 or Li|SPE|NCM811 coin cell tests. SEM images of NCM622. **a** before, and **b** after Li|SPE|NCM622 coin cell cycling test. SEM images of NCM811 **c** before, and **d** after Li|SPE|NCM811 coin cell cycling test. The scale bars are 20 μm. All samples are state of charge of 0%, and for the samples for b and d are after 20 cycles of the coin cell tests under 18 mA g^{-1} (0.30 mA cm^{-2}).

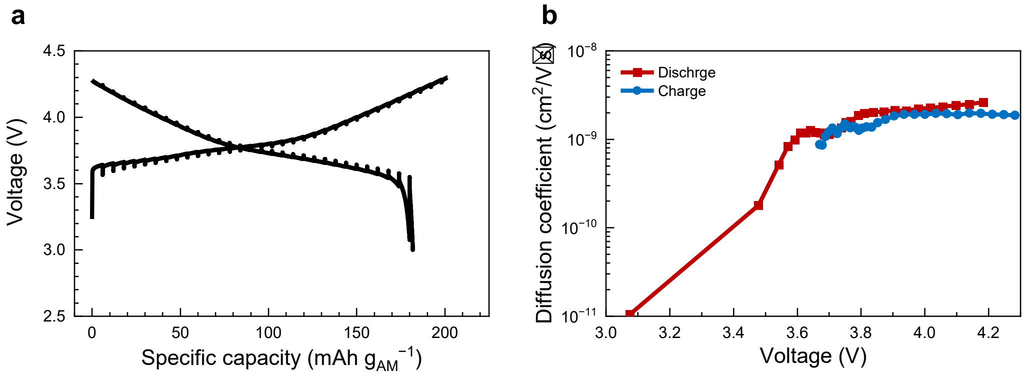

Supplementary Fig. 16: Galvanostatic intermittent titration technique (GITT) result and the calculated diffusivity. a Charge-discharge curve of GITT for Li|SPE|NCM622 coin-cell at 25°C and **b** calculated diffusivity from GITT at each relaxed point.

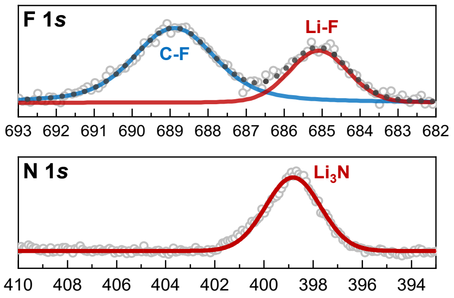

Supplementary Fig. 17: F 1*s* and N 1*s* XPS data of Li-metal negative electrode after full-cell cycling of Li|SPE|NCM622 coin cell. The sample was prepared after 20 cycles of the coin cell tests under 18 mA g^{-1} (0.30 mA cm^{-2}) with state of charge of 0%.

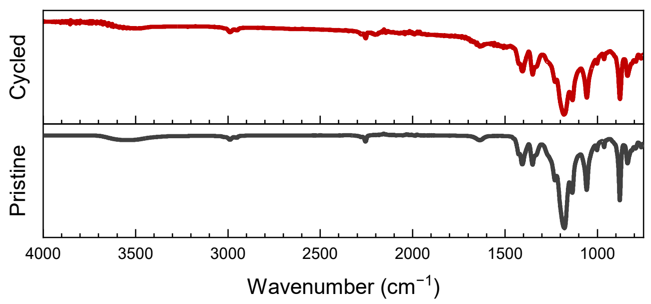

Supplementary Fig. 18: FTIR data of SPE before and after full-cell cycling of Li|SPE|NCM622 coin cell. The sample was prepared after 20 cycles of the coin cell tests under 18 mA g^{-1} (0.30 mA cm^{-2}).

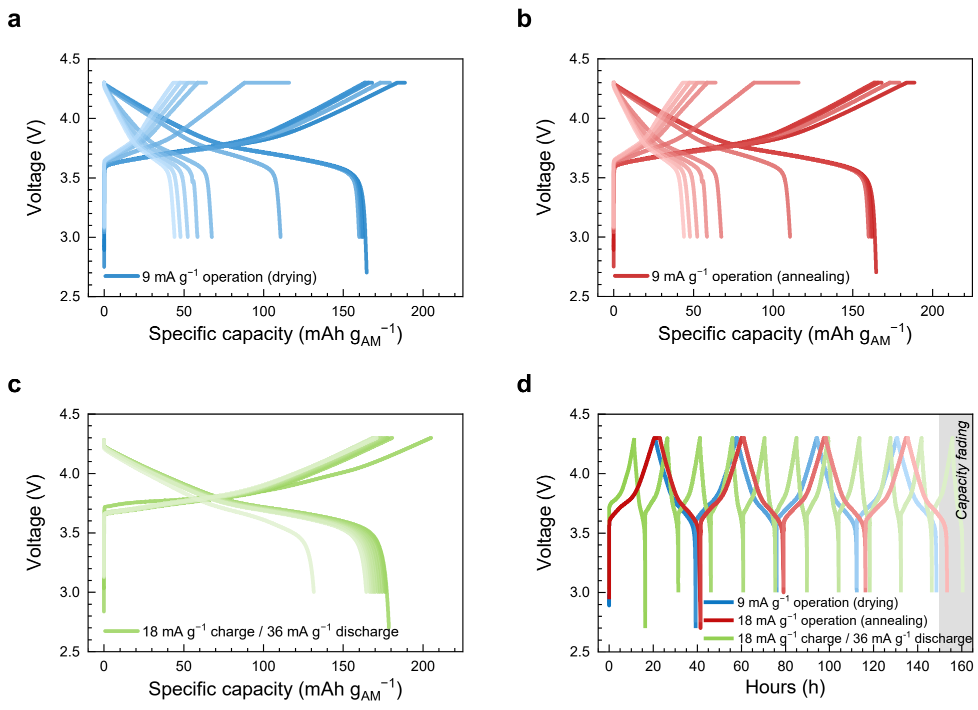

Supplementary Fig. 19: Cycle performance of Li|SPE|NCM622 coin cells under different specific current protocols. 9 mA g^{-1} operation at 45°C with **a** drying and **b** annealing DI water of washed positive electrode, **c** 18 mA g^{-1} charge / 36 mA g^{-1} discharge at 25°C with drying positive electrode, and **d** electrochemical voltage vs. time profiles during cycling of three cases before the sudden capacity fading.

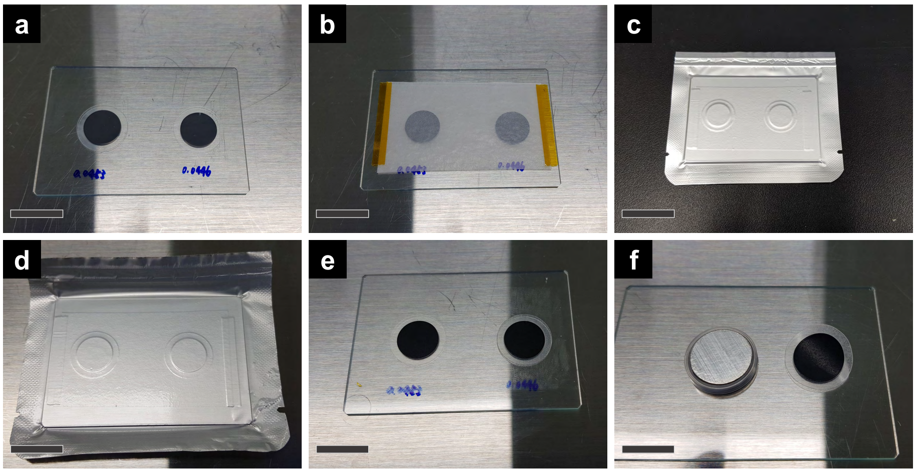

Supplementary Fig. 20: Fabrication process of membrane-electrode assembly (MEA) using WIP method. a Laminated positive electrode and SPE membrane. **b** covered with parchment paper to avoid fluttering during vacuum sealing. Vacuum-packed **c** before and **d–e** after WIP. **f** membrane-electrode assembly (positive electrode-SPE-Li metal negative electrode). The scale bar represents 2 cm.

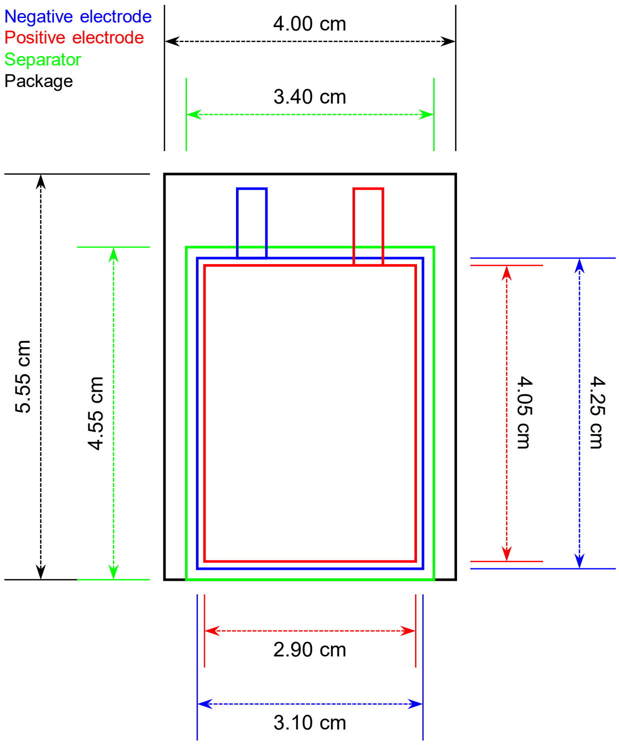

Supplementary Fig. 21: The scheme of positive electrode-SPE-negative electrode with different sizes. The negative electrode is larger than the positive electrode because the negative electrode should take lithium ions from the positive electrode during the charging. SPE is larger than the electrodes to prevent the edge shortening.

Supplementary Table 1. Parameters for SSB pouch cell targeting specific energy of 200 Wh kg^{-1}.

**Electrode**	**Negative**		**Positive**		**Electrolyte**		
Active material	Li		NCM622		True density	0.00	g/cm^3
		μm	10.00	μm	Excess ratio	0.00	vol%
	100.00	wt%	93.00	wt%	E/C ratio	0.00	g/Ah
	100.00	vol%	83.71	vol%	Volume	0.00	cm^3
Electrolyte			PVDF-HFP/LiTFSI/SN		Weight	0.00	g
	0.00	wt%	5.00	wt%	**Separator**	**PVDF-HFP/LiTFSI/SN**	
	0.00	vol%	11.90	vol%	True density	1.80	g/cm^3
Binder					Pore volume	0.00	cm^3
	0.00	wt%	0.00	wt%	Porosity	0.00	vol%
	0.00	vol%	0.00	vol%	Areal ionic bulk resistance	8.20	Ω cm^2
Conducting agent	Super P		Super P		Thickness	82.00	μm
	0.00	wt%	2.00	wt%	Edge margin	2.00	mm
	0.00	vol%	4.39	vol%	Width	3.40	cm
Initial charge capacity	-	mAh/g	207.74	mAh/g	Height	4.55	cm
	-	mAh/cm^2	3.69	mAh/cm^2	Area	15.47	cm^2
Initial discharge capacity	3860.00	mAh/g	187.14	mAh/g	Volume	0.13	cm^3
	4.12	mAh/cm^2	3.32	mAh/cm^2	Weight	0.23	g
Initial coulombic efficiency	-	%	90.08	%	**Package**	**Al pouch film**	
N/P	2.35				Tab type	Single-side	
Discharge nominal voltage	0.00	V vs. Li/Li$^+$	3.81	V vs. Li/Li$^+$	Tab spacing	12.00	mm
Areal ionic bulk resistance		Ω cm^2	115.74	Ω cm^2	Areal weight	180.00	g/m^2
Areal loading weight	1.07	mg/cm^2	19.09	mg/cm^2	Thickness	110.00	μm
Spatial loading density	0.53	g/cm^3	3.70	g/cm^3	Tab sealing width	10.00	mm
True loading density	0.53	g/cm^3	4.28	g/cm^3	Side sealing width	3.00	mm
Pore volume	0.00	cm^3	0.01	cm^3	Cup edge margin	1.50	mm
Porosity	0.00	vol%	13.64	vol%	Width	3.90	cm
Thickness	20.00	μm	51.59	μm	Height	5.45	cm
Edge margin	0.50	mm	-	mm	Area	21.26	cm^2
Width	3.00	cm	2.90	cm	Volume	0.23	cm^3
Height	4.15	cm	4.05	cm	Weight	0.38	g
Area	12.45	cm^2	11.75	cm^2	**Cell specification**		
Volume	0.02	cm^3	0.06	cm^3	Parallel stack	4	unit cell
Weight	0.01	g	0.22	g	C-rate	0.05	C
**Current collector**	**Cu**		**Al**		Nominal discharge voltage	3.81	V
True density	8.96	g/cm^3	2.70	g/cm^3	Discharge capacity	156.08	mAh
Thickness	0.00	μm	20.00	μm	Ionic bulk resistance	2.64	Ω
Width	3.00	cm	2.90	cm	Width	3.90	cm
Height	4.15	cm	4.05	cm	Height	5.45	cm
Area (+ tab)	0.40	cm^2	12.15	cm^2	Thickness	0.89	mm
Volume	0.00	cm^3	0.02	cm^3	Volume	1.46	cm^3
Weight	0.00	g	0.07	g	Weight	2.92	g
**Lead tab**	**Ni tab**		**Al tab**		**Specific power**	**10.18**	**W/kg**
Width	4.00	mm	4.00	mm	**Power density**	**20.39**	**W/L**
Height	10.00	mm	10.00	mm	**Specific energy**	**203.64**	**Wh/kg**
Weight	98.30	mg	36.90	mg	**Energy density**	**407.71**	**Wh/L**

Supplementary Table 2. Parameters for SSB pouch cell targeting specific energy of 280 Wh kg^{-1}.

Electrode	Negative		Positive		Electrolyte		
Active material	Li		NCM622		True density	0.00	g/cm^3
		µm	10.00	µm	Excess ratio	0.00	vol%
	100.00	wt%	94.00	wt%	E/C ratio	0.00	g/Ah
	100.00	vol%	85.88	vol%	Volume	0.00	cm^3
Electrolyte			PVDF-HFP/LiTFSI/SN		Weight	0.00	g
	0.00	wt%	4.00	wt%	**Separator**	**PVDF-HFP/LiTFSI/SN**	
	0.00	vol%	9.66	vol%	True density	1.80	g/cm^3
Binder					Pore volume	0.00	cm^3
	0.00	wt%	0.00	wt%	Porosity	0.00	vol%
	0.00	vol%	0.00	vol%	Areal ionic bulk resistance	9.60	Ω cm^2
Conducting agent	Super P		Super P		Thickness	96.00	µm
	0.00	wt%	2.00	wt%	Edge margin	2.00	mm
	0.00	vol%	4.46	vol%	Width	3.40	cm
Initial charge capacity	-	mAh/g	195.33	mAh/g	Height	4.55	cm
	-	mAh/cm^2	4.82	mAh/cm^2	Area	15.47	cm^2
Initial discharge capacity	3860.00	mAh/g	173.16	mAh/g	Volume	0.15	cm^3
	4.12	mAh/cm^2	4.27	mAh/cm^2	Weight	0.27	g
Initial coulombic efficiency	-	%	88.65	%	**Package**	**Al pouch film**	
N/P	2.09				Tab type	Single-side	
Discharge nominal voltage	0.00	V vs. Li/Li$^+$	3.75	V vs. Li/Li$^+$	Tab spacing	12.00	mm
Areal ionic bulk resistance		Ω cm^2	207.88	Ω cm^2	Areal weight	180.00	g/m^2
Areal loading weight	1.07	mg/cm^2	26.23	mg/cm^2	Thickness	110.00	µm
Spatial loading density	0.53	g/cm^3	3.70	g/cm^3	Tab sealing width	10.00	mm
True loading density	0.53	g/cm^3	4.35	g/cm^3	Side sealing width	3.00	mm
Pore volume	0.00	cm^3	0.01	cm^3	Cup edge margin	1.50	mm
Porosity	0.00	vol%	14.92	vol%	Width	3.90	cm
Thickness	20.00	µm	70.90	µm	Height	5.45	cm
Edge margin	0.50	mm	-	mm	Area	21.26	cm^2
Width	3.00	cm	2.90	cm	Volume	0.23	cm^3
Height	4.15	cm	4.05	cm	Weight	0.38	g
Area	12.45	cm^2	11.75	cm^2	**Cell specification**		
Volume	0.02	cm^3	0.08	cm^3	Parallel stack	20	unit cell
Weight	0.01	g	0.31	g	C-rate	0.05	C
**Current collector**	**Cu**		**Al**		Nominal discharge voltage	3.75	V
True density	8.96	g/cm^3	2.70	g/cm^3	Discharge capacity	1002.93	mAh
Thickness	0.00	µm	20.00	µm	Ionic bulk resistance	0.93	Ω
Width	3.00	cm	2.90	cm	Width	3.90	cm
Height	4.15	cm	4.05	cm	Height	5.45	cm
Area (+ tab)	0.40	cm^2	12.15	cm^2	Thickness	4.18	mm
Volume	0.00	cm^3	0.02	cm^3	Volume	6.28	cm^3
Weight	0.00	g	0.07	g	Weight	13.38	g
**Lead tab**	**Ni tab**		**Al tab**		**Specific power**	**14.06**	**W/kg**
Width	4.00	mm	4.00	mm	**Power density**	**29.96**	**W/L**
Height	10.00	mm	10.00	mm	**Specific energy**	**281.19**	**Wh/kg**
Weight	98.30	mg	36.90	mg	**Energy density**	**599.24**	**Wh/L**

Supplementary Table 3. Parameters for SSB pouch cell targeting specific energy of 310 Wh kg^{-1}.

**Electrode**	**Negative**		**Positive**	
Active material	Li		NCM622	
		µm	10.00	µm
	100.00	wt%	94.00	wt%
	100.00	vol%	85.88	vol%
Electrolyte			PVDF-HFP/LiTFSI/SN	
	0.00	wt%	4.00	wt%
	0.00	vol%	9.66	vol%
Binder	0.00	wt%	0.00	wt%
	0.00	vol%	0.00	vol%
Conducting agent	Super P		Super P	
	0.00	wt%	2.00	wt%
	0.00	vol%	4.46	vol%
Initial charge capacity	-	mAh/g	209.35	mAh/g
	-	mAh/cm^2	6.05	mAh/cm^2
Initial discharge capacity	3860.00	mAh/g	181.21	mAh/g
	4.12	mAh/cm^2	5.24	mAh/cm^2
Initial coulombic efficiency	-	%	86.56	%
N/P			1.94	
Discharge nominal voltage	0.00	V vs. Li/Li$^+$	3.76	V vs. Li/Li$^+$
Areal ionic bulk resistance		Ω cm^2	250.38	Ω cm^2
Areal loading weight	1.07	mg/cm^2	30.74	mg/cm^2
Spatial loading density	0.53	g/cm^3	3.60	g/cm^3
True loading density	0.53	g/cm^3	4.35	g/cm^3
Pore volume	0.00	cm^3	0.02	cm^3
Porosity	0.00	vol%	17.22	vol%
Thickness	20.00	µm	85.39	µm
Edge margin	0.50	mm	-	mm
Width	3.00	cm	2.90	cm
Height	4.15	cm	4.05	cm
Area	12.45	cm^2	11.75	cm^2
Volume	0.02	cm^3	0.10	cm^3
Weight	0.01	g	0.36	g
**Current collector**	**Cu**		**Al**	
True density	8.96	g/cm^3	2.70	g/cm^3
Thickness	0.00	µm	20.00	µm
Width	3.00	cm	2.90	cm
Height	4.15	cm	4.05	cm
Area (+ tab)	0.40	cm^2	12.15	cm^2
Volume	0.00	cm^3	0.02	cm^3
Weight	0.00	g	0.07	g
**Lead tab**	**Ni tab**		**Al tab**	
Width	4.00	mm	4.00	mm
Height	10.00	mm	10.00	mm
Weight	98.30	mg	36.90	mg

**Electrolyte**		
True density	0.00	g/cm^3
Excess ratio	0.00	vol%
E/C ratio	0.00	g/Ah
Volume	0.00	cm^3
Weight	0.00	g
**Separator**	**PVDF-HFP/LiTFSI/SN**	
True density	1.80	g/cm^3
Pore volume	0.00	cm^3
Porosity	0.00	vol%
Areal ionic bulk resistance	8.40	Ω cm^2
Thickness	84.00	µm
Edge margin	1.50	mm
Width	3.30	cm
Height	4.45	cm
Area	14.69	cm^2
Volume	0.12	cm^3
Weight	0.22	g
**Package**	**Al pouch film**	
Tab type	Single-side	
Tab spacing	12.00	mm
Areal weight	180.00	g/m^2
Thickness	110.00	µm
Tab sealing width	10.00	mm
Side sealing width	3.00	mm
Cup edge margin	1.50	mm
Width	3.90	cm
Height	5.45	cm
Area	21.26	cm^2
Volume	0.23	cm^3
Weight	0.38	g
**Cell specification**		
Parallel stack	8	unit cell
C-rate	0.05	C
Nominal discharge voltage	3.76	V
Discharge capacity	491.99	mAh
Ionic bulk resistance	2.75	Ω
Width	3.90	cm
Height	5.45	cm
Thickness	1.84	mm
Volume	2.84	cm^3
Weight	5.96	g
**Specific power**	**15.51**	**W/kg**
**Power density**	**32.58**	**W/L**
**Specific energy**	**310.13**	**Wh/kg**
**Energy density**	**651.51**	**Wh/L**

Supplementary Table 4. Comparison of pouch-cell performance of SSB between this work and other references.

Ref	AM content	AM ratio	SE ratio	CA ratio	Binder ratio	PE loading weight	SE loading weight	NE loading weight	Total cell loading weight	Specific discharge capacity	Nominal discharge voltage	Areal discharge capacity	Specific energy
	mg cm^{-2}	wt%	wt%	wt%	wt%	mg cm^{-2}	mg cm^{-2}	mg cm^{-2}	mg cm^{-2}	mAh g^{-1}	V	mAh cm^{-2}	Wh kg^{-1}
5	5.15	68.6	29.4	2.0	0.0	7.50	151.00	5.30	163.80	125.0	3.59	0.64	14.10
6	6.23	70.0	30.0	0.0	0.0	8.90	127.40	4.30	140.60	131.0	3.76	0.82	21.83
7	3.77	37.7	56.6	5.7	0.0	10.00	200.00	39.00	249.00	120.0	3.85	0.45	6.99
8	5.89	78.5	21.5	0.0	0.0	7.50	150.70	29.40	187.60	130.0	3.92	0.77	15.99
9	1.38	60.0	35.0	5.0	0.0	2.30	90.40	3.00	95.70	100.0	3.28	0.14	4.73
10	7.00	70.0	25.0	2.5	2.5	10.00	90.40	5.30	105.70	168.0	3.7	1.18	41.17
11	29.30	79.2	19.5	1.3	0.0	37.00	7.50	27.00	71.50	120.0	3.57	3.52	175.58
11	14.30	68.1	29.2	1.3	1.4	21.00	4.90	13.00	38.90	110.0	3.67	1.57	148.41
12	15.93	76.2	19.0	1.9	2.9	20.90	13.90	21.50	56.30	122.0	3.51	1.94	121.13
13	9.50	66.0	28.3	2.8	2.8	14.40	11.30	10.20	35.90	111.0	3.62	1.05	106.38
14	4.86	60.0	34.0	6.0	0.0	8.10	49.80	6.00	63.90	110.0	3.71	0.53	31.04
15	115.29	61.0	36.0	3.0	0.0	189.00	18.00	112.00	319.00	123.0	3.75	14.18	166.70
16	6.78	60.0	35.0	5.0	0.0	11.30	52.70	11.30	75.30	121.0	3.59	0.82	39.11
17	10.01	86.3	11.0	1.8	0.9	11.60	134.60	6.00	152.20	119.0	3.69	1.19	28.88
18	34.30	70.0	30.0	0.0	0.0	49.00	133.00	81.00	263.00	89.0	2.11	3.05	24.49
19	0.60	30.0	60.0	10.0	0.0	2.00	75.00	5.00	82.00	1640.0	1.94	0.98	23.28
20	3.60	40.0	50.0	10.0	0.0	9.00	180.00	5.00	194.00	501.0	1.25	1.80	11.62
21	5.72	45.0	50.0	5.0	0.0	12.70	191.10	5.00	208.80	550.0	1.45	3.14	21.83
22	9.90	45.0	25.0	15.0	15.0	22.00	101.00	18.00	141.00	102.0	1.13	1.01	8.09
23	7.00	50.0	50.0	0.0	0.0	14.00	263.00	11.00	288.00	116.0	3.91	0.81	11.02
24	60.75	75.0	15.0	10.0	0.0	81.00	15.00	5.00	101.00	155.0	3.11	9.42	289.95
25	6.00	60.0	30.0	10.0	0.0	10.00	23.00	11.00	44.00	165.0	3.68	0.99	82.80
26	11.70	90.0	10.0	0.0	0.0	13.00	24.00	5.00	42.00	136.0	3.85	1.59	145.86
27	7.39	67.2	28.8	2.0	2.0	11.00	16.00	6.00	33.00	139.0	3.73	1.03	116.14
28	14.70	70.0	25.0	5.0	0.0	21.00	3.00	2.00	26.00	165.0	3.27	2.43	305.05
29	5.20	65.0	20.0	15.0	0.0	8.00	2.00	5.00	15.00	152.0	3.4	0.79	179.16
30	4.80	60.0	32.0	8.0	0.0	8.00	8.00	5.00	21.00	162.0	3.38	0.78	125.16
31	29.28	81.3	14.4	2.9	1.4	36.00	4.62	0.27	40.89	215.0	3.75	6.30	577.42
32	21.65	77.3	19.3	2.9	0.5	28.00	107.80	5.00	140.80	200.0	3.75	4.33	115.30
33	10.30	70.0	10.0	10.0	10.0	14.72	2.55	1.87	19.14	210.0	3.75	2.16	423.97
34	47.25	90.0	10.0	0.0	0.0	52.50	178.25	1.35	232.10	95.0	3.75	4.49	72.52
This work (280 Wh kg^{-1})	24.66	94.0	4.0	2.0	0.0	26.23	17.51	1.07	44.81	173.2	3.75	4.27	357.54
This work (310 Wh kg^{-1})	28.90	94.0	4.0	2.0	0.0	30.74	13.61	1.07	45.42	181.21	3.76	5.24	433.57

Supplementary Table 5. 3D digital twin structure information.

AM:SE (exclude CA)		Value	Porosity (%)	Thickness (μm)	Domain size (μm×μm×μm)	Specific capacity (mAh g^{-1})
96:4 (Pouch cell)	Well-percolation	Experiment	14.92	70.9	-	173.16
		Polydispersed PSD	15.16	70.8	48.4×48.4×70.8	-
		Monodispersed PSD	15.23	72.0	48.4×48.4×72.0	-
96:4 (AM rich)	Poor-percolation	Experiment	35.38	51.6	-	165.79
		Polydispersed PSD	35.55	51.6	48×48×51.6	-
	Well-percolation	Experiment	14.00	42.0	-	175.83
		Polydispersed PSD	14.26	42.0	48×48×42	-
		Monodispersed PSD	15.23	42.0	48×48×42	-
80:20 (AM poor)	Poor-percolation	Experiment	26.94	49.2	-	177.15
		Polydispersed PSD	27.05	49.2	50.0×50.0×49.2	-
	Well-percolation	Experiment	15.52	34.2	-	190.26
		Polydispersed PSD	15.27	34.2	55.2×55.2×34.2	-
		Monodispersed PSD	14.74	34.6	55.2×55.2×34.6	-

Supplementary Table 6. Modeling parameters.

Parameters	Value
Intrinsic electronic conductivity of NCM622 at 45°C (S cm^{-1})	9.79×10^{-4}
Intrinsic lithium diffusion coefficient of NCM622 at 45°C (m^2 s^{-1})	1.53×10^{-13}
Maximum lithium concentration of NCM622 (mol m^{-3})	4.92×10^{4}
Intrinsic ionic conductivity of SPE at 45°C (S cm^{-1})	1.25×10^{-3}
Intrinsic lithium diffusion coefficient of SPE at 45°C (m^2 s^{-1})	4.20×10^{-11}
Lithium transference number of SPE	0.90
Lithium concentration of SPE (mol m^{-3})	845

Supplementary Note 1

Calculation of ionic ASR of the particle composing the composite positive electrode

For calculation of the ionic ASR of the particle composing the composite positive electrode, there is the assumption that the particle has a core–shell structure. Here, the core and shell consist of the AM and SE, respectively.

Equation (1):

$$R = \rho \frac{l}{A}$$

(Here, R is the ionic ASR of the shell, ρ is the resistivity of the shell, l is the nominal length of the shell, and A is the cross-sectional area of the shell.)

Equation (2):

$$\mathrm{d}R = \rho \frac{\mathrm{d}l}{\mathrm{d}A} = \rho \frac{r\,\mathrm{d}\theta}{2\pi r\,\sin\theta\,\mathrm{d}r} = \frac{\rho\,\mathrm{d}\theta}{2\pi\,\sin\theta\,\mathrm{d}r}$$

(Here, r is the spherical radius of the shell, θ is the angle from the pole to the compressed edge point at the spherical core.)

Equation (3): integration of $\mathbf{d}\boldsymbol{R}$ from $\boldsymbol{\theta}$ to $\boldsymbol{\pi - \theta}$ (pole-to-pole compression)

$$R = \frac{\rho}{2\pi\,(r_2 - r_1)} \int_{\theta}^{\pi-\theta} \frac{d\theta}{\sin\theta} = \frac{1}{2\pi\sigma\,(r_2 - r_1)} \int_{\theta}^{\pi-\theta} \frac{d\theta}{\sin\theta}$$

(Here, r_2 is the outer radius of the shell, r_1 is the inner radius of the shell, σ is the ionic conductivity of SE. $\mathrm{d}r = r_2 - r_1$ is the thickness of shell, not infinitesimal.)

We should note that the ASR of trace amounts to the near compressed edge (hatched area) was neglected due to the ease of calculations at the spherical coordinate system.

Equation (4):

$$R = \frac{1}{2\pi\sigma\,(r_2 - r_1)} \ln\left(\frac{1 + \cos\theta}{1 - \cos\theta}\right) = \frac{1}{2\pi\sigma\,(r_2 - r_1)} \ln\left(\frac{r_2 + r_2\cos\theta}{r_2 - r_2\cos\theta}\right)$$

$$= \frac{1}{2\pi\sigma\,(r_2 - r_1)} \ln\left(\frac{r_2 + x}{r_2 - x}\right)$$

(Here, $x = r_2\cos\theta$ and $r_2 - r_2\cos\theta$ is the compressed shell thickness, where $0 < \theta < \frac{\pi}{2}$.)

We should note that the ASR of trace amounts to the near compressed edge (hatched area in **Supplementary Fig. 1**) was neglected due to the ease of calculations at the spherical coordinate system.

Supplementary Note 2

The partial dehydrofluorination of PVDF-HFP can improve the mobility of ions, leading to high ionic conductivity, as proven by the appearance of hydroxyl and C=C double bonds in the FTIR data[1] as shown in **Supplementary Fig. 3**.

Supplementary Note 3

The compatibility of the SPE and Li-metal negative electrode was identified by Li symmetric cycling testing (**Supplementary Fig. 4**). The SPE showed stable cycling with Li metal, indicating that it is compatible with a Li-metal negative electrode.

Supplementary Note 4

The critical current density test of symmetric cells of Li|SPE|Li coin cell was performed with different current densities (**Supplementary Fig. 5**). Based on the result, there was a short-circuit at 0.25 C (0.75 mA cm^{-2}). This result can stem from several reasons such as 1) chemically not optimized SPE (due to dehydrofluorination of SPE during cycling) or 2) insufficient interfacial stability between lithium metal and SPE. To solve this problem, further optimization of SPE components in terms of both chemistry and engineering should be studied in future work.

Supplementary Note 5

Calculation of electrode porosity of composite positive electrode

Equation (5):

$$\varphi = \frac{1 - d_{\mathrm{electrode}}}{\frac{w_{\mathrm{AM+SE}}}{\left(\frac{w_{\mathrm{AM}}}{d_{\mathrm{AM}}}\right) + \left(\frac{w_{\mathrm{SE}}}{d_{\mathrm{SE}}}\right)}} \times 100$$

Here, the $\boldsymbol{\varphi}$ is the volumetric porosity of the electrode [vol%], and the $\boldsymbol{d}_{\mathrm{electrode}}$ is the density of the electrode [g cm^{-3}], $\boldsymbol{w}_{\mathrm{AM+SE}}$ is the total weight fraction of the AM and SE [wt%], $\boldsymbol{w}_{\mathrm{AM}}$ is the weight fraction of the AM [wt%], $\boldsymbol{w}_{\mathrm{SE}}$ is the weight fraction of the SE [wt%], $\boldsymbol{d}_{\mathrm{AM}}$ is the true density of the AM [g cm^{-3}], and $\boldsymbol{d}_{\mathrm{SE}}$ is the true density of the SE [g cm^{-3}].

Supplementary Note 6

In **Supplementary Fig. 8, t**he EDS analysis focused on Ni, Co, Mn, and F atoms because NCM622 consists of Ni, Co, and Mn atoms, and PVDF-HFP in the binder consists of F atoms. The Ni, Co, Mn, and F atoms were uniformly distributed in the positive electrode, demonstrating the positive electrode uniformity.

Supplementary Note 7

In the FIB results (**Supplementary Fig. 9**), the positive electrode prepared using the WIP method also showed the lowest electrode porosity with reduced voids. Moreover, according to the X-ray CT analysis of the positive electrodes prepared using different compressing methods, the positive electrode prepared using the WIP method showed the lowest electrode porosity and highest electrode density. In summary, the WIP method is efficient for preparing ideal positive electrodes with low electrode porosity and high electrode density, leading to the formation of uniform and desirable interfacial contact with lowered resistance of the electrode.

Supplementary Note 8

Calculation of the positive electrode IR drop of SSBs

Equation (6):

$$v_{\mathrm{IR\ drop}} = \left(Q_{\text{core-shell}} \times I_{\text{rate}} \times \frac{T_{\text{PE}}}{2 \times r_{\text{AM}}}\right) \times \left(\text{ASR}_{\text{core-shell}} \times \frac{T_{\text{PE}}}{2 \times r_{\text{AM}}}\right)$$

$$= (J_{\text{core-shell}} \times n_{\text{core-shell}}) \times (\text{ASR}_{\text{core-shell}} \times n_{\text{core-shell}})$$

$$= J_{\text{total}} \times \text{ASR}_{\text{total}}$$

Here, $v_{\mathrm{IR\ drop}}$ is the IR drop [mV]; $Q_{\text{core-shell}}$ [mAh cm^{-2}], $\text{ASR}_{\text{core-shell}}$ [Ohm cm^2], $J_{\text{core-shell}}$, $n_{\text{core-shell}}$ are the areal specific capacity, the areal specific resistance, the current density, and the total number of units in the positive electrode of a core-shell unit building block composed of AM/SE, respectively; I_{rate} is the current rate at which the battery is providing energy [C = 1/h]; T_{PE} [cm] is the thickness of the positive electrode which can be calculated by dividing the areal positive electrode loading by the electrode density; r_{AM} [cm] is the radius of a single particle of AM; J_{total} [mA cm^{-2}] and $\text{ASR}_{\text{total}}$ [Ohm cm^2] are the current density and the areal specific resistance of the positive electrode, respectively.

Equation (7):

$$Q_{\text{core-shell}} = \frac{Q_{\text{AM}} \times d_{\text{AM}} \times V_{\text{AM}}}{A_{\text{core-shell}}}$$

Here, $Q_{\text{core-shell}}$ is the areal specific capacity of a core-shell unit building block composed of AM/SE [mAh cm^{-2}]; Q_{AM} is the specific capacity of the AM [mAh g^{-1}]; d_{AM} is the material true density of the AM [g cm^{-3}]; V_{AM} is the volume of a single particle of AM [cm^3], which can be calculated by **Equation (8)**; and $A_{\text{core-shell}}$ is the area of the composite consisting of the AM and SE [cm^2], which can be calculated using **Equation (9)**.

Equation (8):

$$V_{\mathrm{AM}} = \frac{4}{3}\pi \times (r_{\mathrm{AM}})^3$$

Equation (9):

$$A_{\text{core-shell}} = \pi \times (r_{\mathrm{AM}} + t_{\text{swelled SE}})^2$$

Here, $t_{\text{swelled SE}}$ is the swelled thickness of SE after compression [cm].

Equation (10):

$$\mathrm{ASR} = \frac{\ln \frac{(r_{\mathrm{AM}} + t_{\text{swelled SE}}) + r_{\mathrm{AM}}}{(r_{\mathrm{AM}} + t_{\text{swelled SE}}) - r_{\mathrm{AM}}}}{(4 \times t_{\text{swelled SE}} \times \sigma_{\mathrm{SE}}) \times (r_{\mathrm{AM}} + t_{\text{swelled SE}})^2}$$

Here, σ_{SE} is the ionic conductivity of the SE [S/cm].

Supplementary Note 9

Calculation of electrode specific energy of SSBs

To calculate the electrode specific energy considering only the positive electrode side, which consists of the positive electrode and Al current collector, **Equation (11)** was used.

Equation (11):

$$\varepsilon_{\mathrm{electrode}} = \frac{2 \times Q_{\mathrm{AM}} \times w_{\mathrm{AM}} \times L_{\mathrm{PE}} \times V_{\mathrm{PE}}}{\left(2 \times L_{\mathrm{PE}} + \frac{d_{\mathrm{Al}} \times T_{\mathrm{Al}}}{10}\right)}$$

Here, $\varepsilon_{\mathrm{electrode}}$ is the specific energy of the electrode [Wh kg^{-1}]; Q_{AM} is the discharging capacity of the AM [Ah kg^{-1}]; w_{AM} is the weight ratio of the AM [wt%]; L_{PE} is the areal total loading of the positive electrode [kg cm^{-2}], which can be calculated by multiplying the positive electrode loading thickness by the electrode density; V_{PE} is the voltage of positive electrode vs. Li/Li^{+}; d_{Al} is the density of the Al current collector [kg cm^{-3}]; and T_{Al} is the thickness of the Al current collector [cm].

To calculate the electrode specific energy considering both the positive electrode and negative electrode sides using graphite as the negative electrode material, **Equation (12) and Equation (13)** were used.

Equation (12):

$$\varepsilon_{\mathrm{electrode}} = \frac{2 \times Q_{\mathrm{AM}} \times w_{\mathrm{AM}} \times L_{\mathrm{PE}} \times \left(V_{\mathrm{PE}} - V_{\mathrm{graphite}}\right)}{\left(2 \times L_{\mathrm{PE}} + \frac{d_{\mathrm{Al}} \times T_{\mathrm{Al}}}{10}\right) + \left(2 \times L_{\mathrm{graphite}} + \frac{d_{\mathrm{Cu}} \times T_{\mathrm{Cu}}}{10}\right)}$$

Here, L_{graphite} is the areal total loading of graphite [kg cm^{-2}], which can be calculated using **Equation (13)**; V_{graphite} is the voltage of graphite vs. Li/Li^{+}; d_{Cu} is the density of the Cu current collector [kg cm^{-3}]; and T_{Cu} is the thickness of the Cu current collector [cm].

Equation (13):

$$L_{\mathrm{graphite}} = \frac{Q_{\mathrm{AM}}}{Q_{\mathrm{graphite}}} \times \mathrm{N/P\ ratio} \times L_{\mathrm{PE}}$$

Here, Q_{AM} is the discharging capacity of the AM [Ah kg^{-1}], Q_{graphite} is the discharging capacity of graphite [Ah kg^{-1}], and N/P ratio is the areal capacity ratio of the negative to positive electrode.

To calculate the electrode specific energy considering both the positive electrode and negative electrode sides using Li metal instead of graphite as the negative electrode material, **Equation (14)** was used.

Equation (14):

$$\varepsilon_{\mathrm{electrode}} = \frac{2 \times Q_{\mathrm{AM}} \times w_{\mathrm{AM}} \times L_{\mathrm{PE}} \times V_{\mathrm{PE}}}{\left(2 \times L_{\mathrm{PE}} + \frac{d_{\mathrm{Al}} \times T_{\mathrm{Al}}}{10}\right) + \left(2 \times L_{\mathrm{Li}} + \frac{d_{\mathrm{Cu}} \times T_{\mathrm{Cu}}}{10}\right)}$$

Here, L_{Li} is the areal total loading of Li [kg cm^{-2}].

To calculate the electrode specific energy considering both the positive electrode and negative electrode sides using Li metal as the negative electrode material except with a Cu current collector, **Equation (15)** was used.

Equation (15):

$$\varepsilon_{\mathrm{electrode}} = \frac{2 \times Q_{\mathrm{AM}} \times w_{\mathrm{AM}} \times L_{\mathrm{PE}} \times V_{\mathrm{PE}}}{\left(2 \times L_{\mathrm{PE}} + \frac{d_{\mathrm{Al}} \times T_{\mathrm{Al}}}{10}\right) + (2 \times L_{\mathrm{Li}})}$$

Supplementary Note 10

In **Supplementary Fig. 10**, the SPE membrane with 40 μm thickness was tested as an SE separator for Li|SPE|NCM622 coin cell cycling to enhance the cell-level specific energy. However, the cell-shortening occurred during the initial charge, and it was observed the uneven contact between the SE membrane and the positive electrode with lithium dendrite formation after the cell disassembly. So MEA without lithium metal negative electrode was attacked via WIP until 550 MPa, and it was confirmed that the charge voltage profile was relatively stabilized, but still had the cell shortening with tiny lithium dendrites. Therefore, it was speculated that the uniformly high pressure was not the solution and the thin SE membrane was insufficient to accommodate the high areal capacity of around 4 mAh cm^{-2} of lithium electroplating due to the inhomogeneous charge-transfer. The further thickened SE membrane up to 80 μm had a stable electrochemical cycling profile without cell shortening. The thickness of the SE membrane is quite crucial to the specific energy of SSBs as plotted in **Fig. 5i**, but those should be carefully designed depending on the amount of charge per unit area of the thick positive electrode.

Supplementary Note 11

Calculation of specific energy considering the electrode and SE of SSBs

To calculate the electrode specific energy considering the electrode and SE, **Equation (16)** was used.

Equation (16):

$$\varepsilon_{\mathrm{electrode+SE}} = \frac{2 \times Q_{\mathrm{AM}} \times w_{\mathrm{AM}} \times L_{\mathrm{PE}} \times V_{\mathrm{PE}}}{\left(2 \times L_{\mathrm{PE}} + \frac{d_{\mathrm{Al}} \times T_{\mathrm{Al}}}{10}\right) + \left(2 \times L_{\mathrm{Li}} + \frac{d_{\mathrm{Cu}} \times T_{\mathrm{Cu}}}{10}\right) + \left(2 \times \frac{d_{\mathrm{SE}} \times T_{\mathrm{SE}}}{10}\right)}$$

Here, $\varepsilon_{\mathrm{electrode+SE}}$ is the specific energy of the electrode and SE [Wh kg^{-1}]; Q_{AM} is the discharging capacity of the AM [Ah kg^{-1}]; w_{AM} is the weight ratio of the AM [wt%]; L_{PE} is the areal total loading of the positive electrode [kg cm^{-2}]; L_{Li} is the areal total loading of Li [kg cm^{-2}]; V_{PE} is the voltage of positive electrode vs. Li/Li^{+}; d_{Al} is the density of the Al current collector [kg cm^{-3}]; T_{Al} is the thickness of the Al current collector [cm]; d_{Cu} is the density of the Cu current collector [kg cm^{-3}]; T_{Cu} is the thickness of the Cu current collector [cm]; d_{SE} is the density of the SE membrane [kg cm^{-3}]; and T_{SE} is the thickness of the SE membrane [cm].

Supplementary Note 12

As shown in **Supplementary Fig. 13**, to evaluate the cell performance of SSBs using the developed SPE and freestanding Li-metal negative electrode design, coin-cell tests were performed to examine the effect of the areal positive electrode loading on the cell performance. The weight ratio of AM was 94 wt%, and the current rate was 0.1 C for all the coin-cell tests. One coin cell had a low positive electrode loading of 3.50 mg cm^{-2}, and the other had a high positive electrode loading of 22.67 mg cm^{-2}.

The results of the cycling test indicated that the areal discharging capacity was 179.2 mAh g_{AM}^{-1} with the low areal positive electrode loading and 174.4 mAh g_{AM}^{-1} with the high areal positive electrode loading, which are similar values. However, the discharging capacity and energy increased from 0.791 mAh and 3.01 mWh with the low areal positive electrode loading to 6.081 mAh and 23.18 mWh with the high areal positive electrode loading. This result indicates that a high areal positive electrode loading is necessary for producing high capacity and energy output of the SSBs.

The coulombic efficiency was similar for the low areal positive electrode loading (99.25%) and high areal positive electrode loading (99.20%). However, the energy efficiency was lower for the coin cell with high areal positive electrode loading (96.70%) than for that with the low areal positive electrode loading (99.25%). This result suggests a trade-off between the energy and energy efficiency depending on the areal positive electrode loading. The higher positive electrode IR drop with higher areal positive electrode loading results in low energy efficiency.

Capacity retention is also an essential parameter in cell performance. The coin cell exhibited stable capacity retention when using low areal positive electrode loading during 20 cycles. However, sudden capacity fading was observed after the 7th cycle for the coin cell using high areal positive electrode loading. This result indicates that a high areal positive electrode loading induces a high IR drop, leading to the high resistance of the cell and capacity decay. The

resistance of the cell before and after full-cell cycling was measured using EIS, and it increased as expected in calculating the positive electrode IR drop tendency depending on the areal positive electrode loading. In summary, both the negative electrode and SPE showed good stability during cycling, and the capacity decay may stem from the increased resistance of the cell due to the high positive electrode IR drop when using high areal positive electrode loading.

Supplementary Note 13

Li|SPE|NCM811 configured coin cells were not well-operated for unspecified reasons. The laminate MEA composed of NCM811 as an AM, the SE membranes turned brownish, the yield rate of those cells was low, and some were not discharged well as shown in this figure. The issues of SE membrane color change were also found in the degraded cell with NCM622 after cycle test in **Supplementary Fig. 10**. We assumed that those were related to dehydrofluorination of PVDF based polymers which is well-known to this chemical under the high pH conditions[2,3] based on the several supporting data. According to this, we also specified that the residual lithium compounds, such as Li_2CO_3 or LiOH on the surface of NCM811, triggered the degradation of SPE and were found to have low electrochemical cycling behavior.

Supplementary Note 14

After the full-cell cycling of the coin cell in **Supplementary Fig. 17**, F 1*s* XPS data of the Li-metal negative electrode side was collected, and formation of C-F and LiF, stemming from the PVDF-HFP in the SPE, was observed. In addition, N 1*s* XPS data of the Li-metal negative electrode after cycling could show the formation of Li_3N, stemming from the SN decomposition, was observed. The LiF and Li_3N formed the stable layer on the Li metal negative electrode[4].

Supplementary Note 15

FTIR spectra of the SPE before and after full-cell cycling were analyzed and indicated no structural change during cycling (**Supplementary Fig. 18**).

Supplementary Note 16

In **Supplementary Fig. 20**, as the first step, the positive electrode and SPE were compressed by the WIP separately. The WIP method can make not only uniform interface formation for the positive electrode but also squeeze and enhance the mechanical strength of SPE. After that, the compressed positive electrode and SPE were laminated and compressed again using the WIP method to maximize the interfacial contact between the positive electrode and SPE. The WIP method consists of three steps. The first step is increasing the pressure to 5500 bar gradually for 5 mins. Second, the pressure was maintained at 5500 bar for 5 mins. Finally, the pressure was reduced to zero gradually for 10 mins. After the WIP, the Li metal negative electrode can be simply put onto the SPE of laminated positive electrode-SPE composite.

Supplementary References

1. Aliahmad, N., Shrestha, S., Varahramyan, K., & Agarwal, M. Poly (vinylidene fluoride-hexafluoropropylene) polymer electrolyte for paper-based and flexible battery applications. *AIP Adv*. **6**, 065206 (2016).
2. Seong, W. M., Cho, K. H., Park, J. W., Park, H., Eum, D., Lee, M. H., Kim, I. S., Lim, J. & Kang, K. Controlling residual lithium in high-nickel (> 90%) lithium layered oxides for cathodes in lithium-ion batteries. *Angew. Chem. Int. Ed*. **59**, 18662-18669 (2020).
3. Taguet, A., Ameduri, B. & Boutevin, B. Crosslinking of vinylidene fluoride-containing fluoropolymers. In: Crosslinking in Materials Science. *Adv. Polym. Sci*. **184**, 127-211 (2005).
4. Zhang, Z., Wang, J., Zhang, S., Ying, H., Zhuang, Z., Ma, F., Huang, P., Yang, T., Han, G. & Han, W. Q. Stable all-solid-state lithium metal batteries with Li_3N-LiF-enriched interface induced by lithium nitrate addition. *Energy Storage Mater*. **43**, 229-237 (2021).
5. Whiteley, J. M., Woo, J. H., Hu, E., Nam, K. W. & Lee, S. H. Empowering the lithium metal battery through a silicon-based superionic conductor. *J. Electrochem. Soc*. **161**, A1812 (2014).
6. Zhang, Z., Chen, S., Yang, J., Wang, J., Yao, L., Yao, X., Cui, P. & Xu, X. Interface re-engineering of $Li_{10}GeP_2S_{12}$ electrolyte and lithium anode for all-solid-state lithium batteries with ultralong cycle life. *ACS Appl. Mater. Interfaces* **10**, 2556-2565 (2018).
7. Woo, J. H., Trevey, J. E., Cavanagh, A. S., Choi, Y. S., Kim, S. C., George, S. M., Oh, K. H. & Lee, S. H. Nanoscale interface modification of $LiCoO_2$ by Al_2O_3 atomic layer deposition for solid-state Li batteries. *J. Electrochem. Soc*. **159**, A1120 (2012).
8. Xie, D., Chen, S., Zhang, Z., Ren, J., Yao, L., Wu, L., Yao, X. & Xu, X. High ion conductive Sb_2O_5-doped β-Li_3PS_4 with excellent stability against Li for all-solid-state

lithium batteries. *J. Power Sources* **389**, 140-147 (2018).

9. Ulissi, U., Agostini, M., Ito, S., Aihara, Y. & Hassoun, J. All solid-state battery using layered oxide cathode, lithium-carbon composite anode and thio-LISICON electrolyte. *Solid State Ion*. **296**, 13-17 (2016).
10. Choi, S. J., Choi, S. H., Bui, A. D., Lee, Y. J., Lee, S. M., Shin, H. C. & Ha, Y. C. LiI-doped sulfide solid electrolyte: enabling a high-capacity slurry-cast electrode by low-temperature post-sintering for practical all-solid-state lithium batteries. *ACS Appl. Mater. Interfaces* **10**, 31404-31412 (2018).
11. Nam, Y. J., Oh, D. Y., Jung, S. H. & Jung, Y. S. Toward practical all-solid-state lithium-ion batteries with high energy density and safety: Comparative study for electrodes fabricated by dry-and slurry-mixing processes. *J. Power Sources* **375**, 93-101 (2018).
12. Yamamoto, M., Terauchi, Y., Sakuda, A. & Takahashi, M. Binder-free sheet-type all-solid-state batteries with enhanced rate capabilities and high energy densities. *Sci. Rep.* **8**, 1212 (2018).
13. Sakuda, A., Kuratani, K., Yamamoto, M., Takahashi, M., Takeuchi, T. & Kobayashi, H. All-solid-state battery electrode sheets prepared by a slurry coating process. *J. Electrochem. Soc*. **164**, A2474 (2017).
14. Kato, Y., Hori, S., Saito, T., Suzuki, K., Hirayama, M., Mitsui, A., Yonemura, M., Iba, H. & Kanno, R. High-power all-solid-state batteries using sulfide superionic conductors. *Nat. Energy* **1**, 16030 (2016).
15. Kato, Y., Shiotani, S., Morita, K., Suzuki, K., Hirayama, M. & Kanno, R. All-solid-state batteries with thick electrode configurations. *J. Phys. Chem. Lett*. **9**, 607-613 (2018).
16. Ito, S., Fujiki, S., Yamada, T., Aihara, Y., Park, Y., Kim, T. Y., Baek, S. W., Lee, J. M., Doo, S. & Machida, N. A rocking chair type all-solid-state lithium ion battery adopting Li_2O–ZrO_2 coated $LiNi_{0.8}Co_{0.15}Al_{0.05}O_2$ and a sulfide based electrolyte. *J. Power Sources*

248, 943-950 (2014).

17. Kim, D. H., Oh, D. Y., Park, K. H., Choi, Y. E., Nam, Y. J., Lee, H. A., Lee, S. M. & Jung, Y. S. Infiltration of solution-processable solid electrolytes into conventional Li-ion-battery electrodes for all-solid-state Li-ion batteries. *Nano Lett*. **17**, 3013-3020 (2017).
18. Kraft, M. A., Ohno, S., Zinkevich, T., Koerver, R., Culver, S. P., Fuchs, T., Senyshyn, A., Indris, S., Morgan, B. J. & Zeier, W. G. Inducing high ionic conductivity in the lithium superionic argyrodites $Li_{6+x}P_{1-x}Ge_xS_5I$ for all-solid-state batteries. *J. Am. Chem. Soc.* **140**, 16330-16339 (2018).
19. Yamada, T., Ito, S., Omoda, R., Watanabe, T., Aihara, Y., Agostini, M., Ulissi, U., Hassoun, J. & Scrosati, B. All solid-state lithium–sulfur battery using a glass-type P_2S_5–Li_2S electrolyte: benefits on anode kinetics. *J. Electrochem. Soc*. **162**, A646 (2015).
20. Yao, X., Liu, D., Wang, C., Long, P., Peng, G., Hu, Y. S., Li, H., Chen, L. & Xu, X. High-energy all-solid-state lithium batteries with ultralong cycle life. *Nano Lett.* **16**, 7148-7154 (2016).
21. Zhang, Q., Peng, G., Mwizerwa, J. P., Wan, H., Cai, L., Xu, X. & Yao, X. Nickel sulfide anchored carbon nanotubes for all-solid-state lithium batteries with enhanced rate capability and cycling stability. *J. Mater. Chem. A* **6**, 12098-12105 (2018).
22. Yu, S., Mertens, A., Tempel, H., Schierholz, R., Kungl, H. & Eichel, R. A. Monolithic all-phosphate solid-state lithium-ion battery with improved interfacial compatibility. *ACS Appl. Mater. Interfaces* **10**, 22264-22277 (2018).
23. Finsterbusch, M., Danner, T., Tsai, C. L., Uhlenbruck, S., Latz, A. & Guillon, O. High capacity garnet-based all-solid-state lithium batteries: fabrication and 3D-microstructure resolved modeling. *ACS Appl. Mater. Interfaces* **10**, 22329-22339 (2018).
24. Chen, R. J., Zhang, Y. B., Liu, T., Xu, B. Q., Lin, Y. H., Nan, C. W. & Shen, Y.

Addressing the interface issues in all-solid-state bulk-type lithium ion battery via an all-composite approach. *ACS Energy Lett*. **9**, 9654-9661 (2017).

25. Park, M. S., Jung, Y. C. & Kim, D. W. Hybrid solid electrolytes composed of poly (1, 4-butylene adipate) and lithium aluminum germanium phosphate for all-solid-state Li/$LiNi_{0.6}Co_{0.2}Mn_{0.2}O_2$ cells. *Solid State Ion*. **315**, 65-70 (2018).
26. Wakayama, H., Yonekura, H. & Kawai, Y. Three-dimensional bicontinuous nanocomposite from a self-assembled block copolymer for a high-capacity all-solid-state lithium battery cathode. *Chem. Mater*. **28**, 4453-4459 (2016).
27. Ates, T., Keller, M., Kulisch, J., Adermann, T. & Passerini, S. Development of an all-solid-state lithium battery by slurry-coating procedures using a sulfidic electrolyte. *Energy Storage Mater.* **17**, 204–210 (2019).
28. Hovington, P., Lagacé, M., Guerfi, A., Bouchard, P., Mauger, A., Julien, C. M., Armand, M. & Zaghib, K. New lithium metal polymer solid state battery for an ultrahigh energy: nano C-$LiFePO_4$ versus nano $Li_{1.2}V_3O_8$. *Nano Lett*. **15**, 2671-2678 (2015).
29. Porcarelli, L., Aboudzadeh, M. A., Rubatat, L., Nair, J. R., Shaplov, A. S., Gerbaldi, C. & Mecerreyes, D. Single-ion triblock copolymer electrolytes based on poly (ethylene oxide) and methacrylic sulfonamide blocks for lithium metal batteries. *J. Power Sources* **364**, 191-199 (2017).
30. Bouchet, R., Maria, S., Meziane, R., Aboulaich, A., Lienafa, L., Bonnet, J. P., Phan, N. T., Bertin, D., Gigmes, D., Devaux, D., Denoyel, R. & Armand, M. Single-ion BAB triblock copolymers as highly efficient electrolytes for lithium-metal batteries. *Nat. Mater*. **12**, 452-457 (2013).
31. Lee, Y. G., Fujiki, S., Jung, C., Suzuki, N., Yashiro, N., Omoda, R., Ko, D. S., Shiratsuchi, T., Sugimoto, T., Ryu, S., Ku, J. H., Watanabe, T., Park, Y., Aihara, Y., Im, D. & Han, I. T. High-energy long-cycling all-solid-state lithium metal batteries enabled

by silver–carbon composite anodes. *Nat. Energy* **5**, 299-308 (2020).

32. Tan, D. H., Chen, Y. T., Yang, H., Bao, W., Sreenarayanan, B., Doux, J. M., Li, W., Lu, B., Ham, S. Y., Sayahpour, B., Scharf, J., Wu, E. A., Deysher, G., Han, H. E., Hah, H. J., Jeong, H., Lee, J., Chen, Z. & Meng, Y. S. Carbon-free high-loading silicon anodes enabled by sulfide solid electrolytes. *Science* **373**, 1494-1499 (2021).
33. Lee, M. J., Han, J., Lee, K., Lee, Y. J., Kim, B. G., Jung, K. N., Kim, B. J. & Lee, S. W. Elastomeric electrolytes for high-energy solid-state lithium batteries. *Nature* **601**, 217-222 (2022).
34. Zhou, L., Zuo, T. T., Kwok, C. Y., Kim, S. Y., Assoud, A., Zhang, Q., Janek, J. & Nazar, L. F. High areal capacity, long cycle life 4 V ceramic all-solid-state Li-ion batteries enabled by chloride solid electrolytes. *Nat. Energy* **7**, 83-93 (2022).
